# Effects of Tobacco-Smoking

**Published:** 1874-11

**Authors:** 


### EFFECTS OF TOBACCO-SMOKING.

Omitting all details of analysis of different tobaccos as too familiar for repetition, my experiments have led me to conclude—

1. That nicotine is the special agent concerned in vital paralysis and in disturbances of muscular co-ordination, and that its action upon the medullary centers is propagated by way of the pneumogastric nerve; that the cerebella centres (co-ordinating the muscles concerned in locomotion), and the corpora striata (or great motor ganglia of the cerebrum), are next affected; in other words, that the motor tracts follow the vital in yielding to the influence of the poison.

2. That the cortex of the brain is the last to be affected by nicotine, but is more specifically affected by the pyrieline, picoline, and collidine bases. Hence the difference in physiological action between Honradez, with its minimum of nicotine, and perique and cavendish, with their excess; also the analogous difference between Havana cigars and cigars manufactured from Connecticut leaf.

3. That smoking is often the exciting cause of the various neuroses, and always a fruitful source of local aneurism, by impairing the nervous circulation and laying the foundation for defective nutrition in various directions. Cessation from tobacco should be made a condition precedent to medical treatment in writer's cramp and nervous affections of that type, (the paralytic.)

I am not going to take any radical ground on the tobacco question in its general aspects. Every man must judge for himself and experiment for himself, as to the physiological action of the weed. I have simply recorded my own experiences and experiments, and the conclusions to which they have impelled me. I will not even say that I shall never smoke another cigar, for temptations are often strong and sudden; but I will say that, in such an event, I should regard myself as the victim of a nervous infirmity, not as one merely indulging himself in a harmless and pleasant luxury—of a devil far easier to get out of the bottle, to apply a Moslem legend, than to get back and cork in again.—*Popular Science Monthly for November.*

